# Targeting FXYD2 by cardiac glycosides potently blocks tumor growth in ovarian clear cell carcinoma

**DOI:** 10.18632/oncotarget.7497

**Published:** 2016-02-19

**Authors:** I-Ling Hsu, Cheng-Yang Chou, Yi-Ying Wu, Jia-En Wu, Chen-Hsien Liang, Yao-Tsung Tsai, Jhen-Yu Ke, Yuh-Ling Chen, Keng-Fu Hsu, Tse-Ming Hong

**Affiliations:** ^1^ Institute of Basic Medical Sciences, National Cheng Kung University Hospital, College of Medicine, National Cheng Kung University, Tainan, Taiwan; ^2^ Department of Obstetrics and Gynecology, National Cheng Kung University Hospital, College of Medicine, National Cheng Kung University, Tainan, Taiwan; ^3^ Graduate Institute of Clinical Medicine, National Cheng Kung University Hospital, College of Medicine, National Cheng Kung University, Tainan, Taiwan; ^4^ Institute of Oral Medicine, National Cheng Kung University Hospital, College of Medicine, National Cheng Kung University, Tainan, Taiwan

**Keywords:** FXYD2, ovarian clear cell carcinoma, Na^+^/K^+^-ATPase, cardiac glycoside

## Abstract

Ovarian clear cell carcinoma (OCCC) is an aggressive neoplasm with a high recurrence rate that frequently develops resistance to platinum-based chemotherapy. There are few prognostic biomarkers or targeted therapies exist for patients with OCCC. Here, we identified that FXYD2, the modulating subunit of Na^+^/K^+^-ATPases, was highly and specifically expressed in clinical OCCC tissues. The expression levels of FXYD2 were significantly higher in advanced-stage of OCCC and positively correlated with patients' prognoses. Silencing of FXYD2 expression in OCCC cells inhibited Na^+^/K^+^-ATPase enzyme activity and suppressed tumor growth via induction of autophagy-mediated cell death. We found that high FXYD2 expression in OCCC was transcriptionally regulated by the transcriptional factor HNF1B. Furthermore, up-regulation of FXYD2 expression significantly increased the sensitivity of OCCC cells to cardiac glycosides, the Na^+^/K^+^-ATPase inhibitors. Two cardiac glycosides, digoxin and digitoxin, had a great therapeutic efficacy in OCCC cells *in vitro* and *in vivo*. Taken together, our results demonstrate that FXYD2 is functionally upregulated in OCCC and may serve as a promising prognostic biomarker and therapeutic target of cardiac glycosides in OCCC.

## INTRODUCTION

Ovarian cancer has the highest mortality rate of all gynecologic cancers in the United States and worldwide because it is difficult to detect before it has progressed to advanced stages [1]. Ovarian cancer is a heterogeneous disease, exhibiting a wide range of morphological characteristics, clinical responses, and genetic alterations, which pose a major clinical challenge for diagnosis and therapeutic strategy. Ovarian clear cell carcinoma (OCCC), a subtype of epithelial ovarian cancers, is considered an aggressive and high-grade neoplasm that spreads rapidly throughout the pelvis [2, 3]. Patients with OCCC have poor prognosis due to high resistance to chemotherapy and high recurrence rate [4]. Therefore, molecular classification of OCCC and the identification of stratifying biomarkers and potential targeted therapies are required for improved prognosis.

Na^+^/K^+^-ATPase, an oligomeric transmembrane protein composed of α, β, and γ subunits, is highly expressed in epithelial cells and plays an important role in the kidney [5, 6]. Na^+^/K^+^-ATPase activity is required to maintain membrane potential homeostasis and has been implicated in many cellular functions and the pathogenesis of specific diseases [7]. Na^+^/K^+^-ATPase upregulation has been found in many cancers [8–12], giving rise to the idea that Na^+^/K^+^-ATPase inhibitors might have the potential to treat cancers. Cardiac glycosides, the Na^+^/K^+^-ATPase inhibitors, were found to possess strong anti-tumor activity [13–15]. A decrease in Na^+^/K^+^-ATPase activity or expression also inhibits cancer cell proliferation and survival [16, 17]. However, the mechanisms that underlie the role of Na^+^/K^+^-ATPase in tumorigenesis remain unclear.

Here, we show that FXYD2, the γ subunit and enzymatic modulator of the Na^+^/K^+^-ATPase [18], is upregulated in OCCC tumors and correlated with OCCC tumorigenesis. Upregulation of FXYD2 in cancer cells has been shown to confer dependence on FXYD2 for survival, and the inhibition of Na^+^/K^+^-ATPase by cardiac glycosides has a great therapeutic efficacy in OCCC cells expressing high levels of FXYD2 through an autophagic effect.

## RESULTS

### FXYD2 is highly and specifically expressed in ovarian clear cell carcinoma

**Figure 1:**
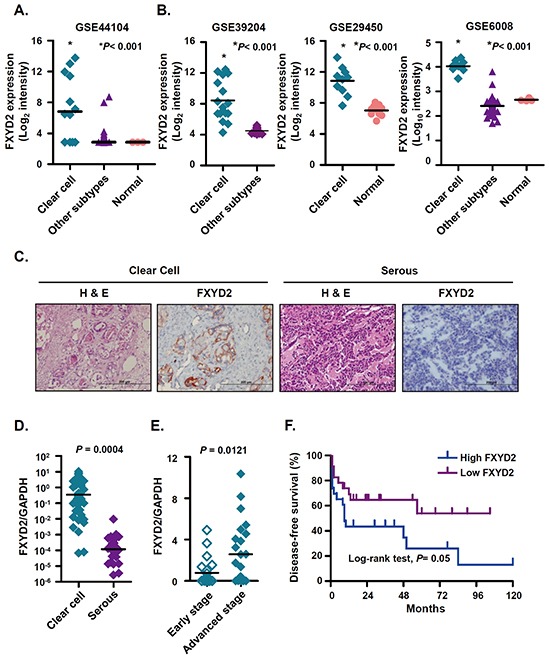
FXYD2 is highly expressed in ovarian clear cell cancer **A.** and **B.** the mRNA expression levels of FXYD2 were compared in clinical ovarian cancer specimens from our Affymetrix GeneChip HG-U133_Plus_2 analysis (GSE44104) and three GEO databases. All of the specimen groups were compared to clear cell ovarian cancer group using one-way ANOVA followed by Bonferroni multiple comparisons test. **C.** representative images of immunohistochemical analysis of FXYD2 in ovarian cancer sections. Consecutive sections were stained with hematoxylin and eosin (H&E) to define representative tumor regions. Magnification ×200. Scale bar, 200 μm. Comparison of FXYD2 mRNA expressions in clinical ovarian cancer specimens (**D.** clear cell, n = 46; serous, n = 28) and (**E.** early, stage 1 and 2, n=22; advanced, stage 3 and 4, n=24). The FXYD2 expression levels were determined by qRT-PCR and normalized to GAPDH expression. All of the qRT-PCR data presented is from three independent experiments that were analyzed using an unpaired *t* test. **F.** Kaplan-Meier survival plots for patients with ovarian clear cell carcinoma (n = 46) according to FXYD2 mRNA expression. The FXYD2 mRNA levels were measured by qRT-PCR and normalized to the GAPDH expression. The median value was used to divide patients into high (n = 23) and low (n = 23) FXYD2 expression groups. Statistical comparison of Kaplan-Meier curve was analyzed by the log-rank test.

To investigate potential biomarkers or therapeutic targets in OCCC, we surveyed global gene expression profiles of ovarian cancers and determined a candidate signature consisting of genes that were highly upregulated in OCCC. In our previous microarray study [19], (GEO number: GSE44104), FXYD2 was ranked as the second highest up-regulated gene in OCCC. Compared with other ovarian cancer subtypes and normal ovarian surface epithelia, FXYD2 was highly expressed in OCCC (Figure 1A). High expression of FXYD2, specifically in OCCC tumors, was also observed in other GEO databases, including GSE39204, GSE29450 and GSE6008 (Figure 1B). High levels of FXYD2 were detected in OCCC but not in normal ovarian tissues or in other subtypes of epithelial ovarian cancers. FXYD proteins are expressed in a tissue- and isoform-specific manner and Na^+^/K^+^-ATPase upregulation was observed across many types of cancers, hence the expression level of each Na^+^/K^+^-ATPase subunit was assessed in various subtypes of ovarian cancers using microarray data. As shown in Supplementary Figure S1, only FXYD2 was observed to be specifically upregulated in OCCC among all the other subunits of the Na^+^/K^+^-ATPase. In addition, compared with the human kidney embryo cell line, HEK293, OCCC cell lines were shown to harbor higher expression levels of FXYD2 (Supplementary Figure S2). Next, we assessed FXYD2 protein and mRNA levels in clinical ovarian cancer specimens using immunohistochemistry and qRT-PCR, respectively. The clinical and pathologic features of patient tumors analyzed by immunohistochemistry and qRT-PCR are summarized in Supplementary Table S1 and S2, respectively. We show that FXYD2 status correlated strongly with clear cell histology in both analyses of clinical ovarian cancer specimens (*P* < 0.0001). Immunohistochemical analysis of 144 ovarian cancer tissues indicated that OCCC samples displayed a significantly higher percentage of cells that stained positive for FXYD2 compared with other ovarian cancer subtypes (Supplementary Table S1), with high FXYD2 expression observed in the membrane (Figure 1C). High FXYD2 expression was also observed by qRT-PCR analysis in OCCC samples (mean: 1.7159, n = 46) compared with serous carcinoma samples (mean: 0.0006, n = 28, *P* = 0.004, Figure 1D). In addition, FXYD2 expression level was significantly higher in advanced-stage disease (stage 3 and 4; mean: 2.9869, n = 24) compared with early tumor stages (stage 1 and 2; mean: 0.8358, n = 22, *P* = 0.0121, Figure 1E). Moreover, stratification of OCCC patients based on FXYD2 mRNA levels (median value Log_2_ ratio = 0.345; FXYD2-high; n = 23, and FXYD2-low; n = 23) revealed that patients with high FXYD2 expression displayed decreased disease-free survival compared with patients with low FXYD2 expression (*P* = 0.05; log-rank test, Figure 1F). Together, our results suggest that FXYD2 may represent a viable prognostic biomarker to use in OCCC subtype classification.

### FXDY2 suppression promotes autophagic cell death and inhibits tumor formation *in vitro* and *in vivo*

**Figure 2:**
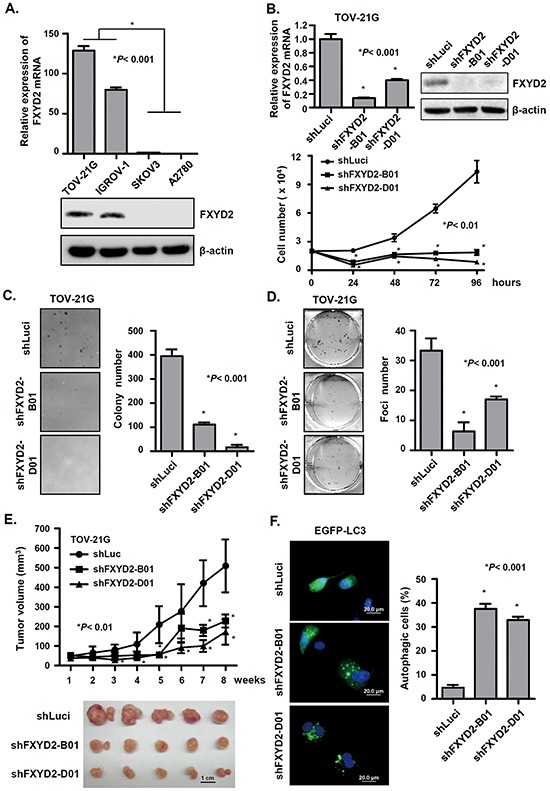
Repression of FXYD2 inhibits OCCC cell growth and tumor formation *in vitro* and *in vivo* **A.** the endogenous expression of FXYD2 in ovarian cancer cell lines was measured by qRT-PCR, normalized to GAPDH expression, and by Western blot using β-actin control. **B.** FXYD2 knockdown inhibits OCCC cell proliferation. TOV-21G cells were infected with lentiviruses carrying shRNA targeting FXYD2 (shFXYD2-B01 and D01) or the negative control luciferase (shLuci). qRT-PCR and Western blotting were used to analyze FXYD2 expression. Cell proliferation was determined by cell number counting. Data from three independent experiments were analyzed using an unpaired *t* test. Anchorage-independent and density-dependent growth of TOV-21G cells infected with shFXYD2 or shLuci viruses was assessed by soft agar assays (**C**) and foci formation assays (**D**) respectively. Data from three independent experiments were analyzed using an unpaired *t* test. **E.** TOV-21G cells were subcutaneously injected into NOD/SCID mice and after one week inoculation the tumor volume was measured every week for two months. The mice were euthanized at 8 weeks post inoculation and tumors were presented in lower panel. **F.** TOV-21G cells infected with shFXYD2 or shLuci viruses were transfected with an EGFP-LC3 plasmid. After 48 h transfection, EGFP-LC3 spots were observed using a fluorescence microscope. Scale bar, 20 μm. Cells with EGFP-LC3 spots represent autophagic cells and were counted among 200 EGFP-positive cells. All experimental groups were replicated three times and analyzed using an unpaired *t* test.

From the clinical finding that FXYD2 was specifically increased in OCCC, we decided to examine FXYD2 expression in OCCC cell lines TOV-21G and IGROV-1, and in non-OCCC cell lines, SKOV3 and A2780. As expected, TOV-21G and IGROV-1 displayed strikingly higher FXYD2 levels compared with SKOV3 and A2780 (Figure 2A). To determine the cellular function of FXYD2, OCCC cells were infected with lentiviral-based shRNAs targeting FXYD2 or control, and cell proliferation was examined. We found that knockdown of FXYD2 significantly suppressed the proliferation of TOV-21G and IGROV-1 cells (Figure 2B and Supplementary Figure S3A). Moreover, silencing FXYD2 in TOV-21G cells also inhibited anchorage-independent cell growth (Figure 2C) and cell transformation, as assessed by foci formation assay (Figure 2D). To assess the *in vivo* functions of FXYD2, TOV-21G cells transduced with shRNA targeting FXYD2 were subcutaneously inoculated into SCID mice, and tumor size was assessed. Suppression of FXYD2 was shown to lead to a significant decrease in tumor growth rate, as well as tumor size *in vivo* (Figure 2E). Mechanistically, the anti-proliferative effects of FXYD2 suppression were not due to changes in the cell cycle or apoptosis (as measured by cleaved-caspase 3 present) (Supplementary Figure S3B and S3C) but were instead mediated by the induction of autophagy as assessed by using the autophagosome marker EGFP-LC3. As shown in Figure 2F, genetic depletion of FXYD2 in OCCC cells led to an increase in the formation of GFP-LC3 puncta, a marker of autophagy, and LC3-ll expression (Supplementary Figure S3C). Together, our results suggest that the suppression of FXYD2 inhibits tumor formation by increasing autophagy activity.

### FXYD2 increases Na^+^/K^+^-ATPase activity and sensitivity of OCCC cells to cardiac glycosides

**Figure 3:**
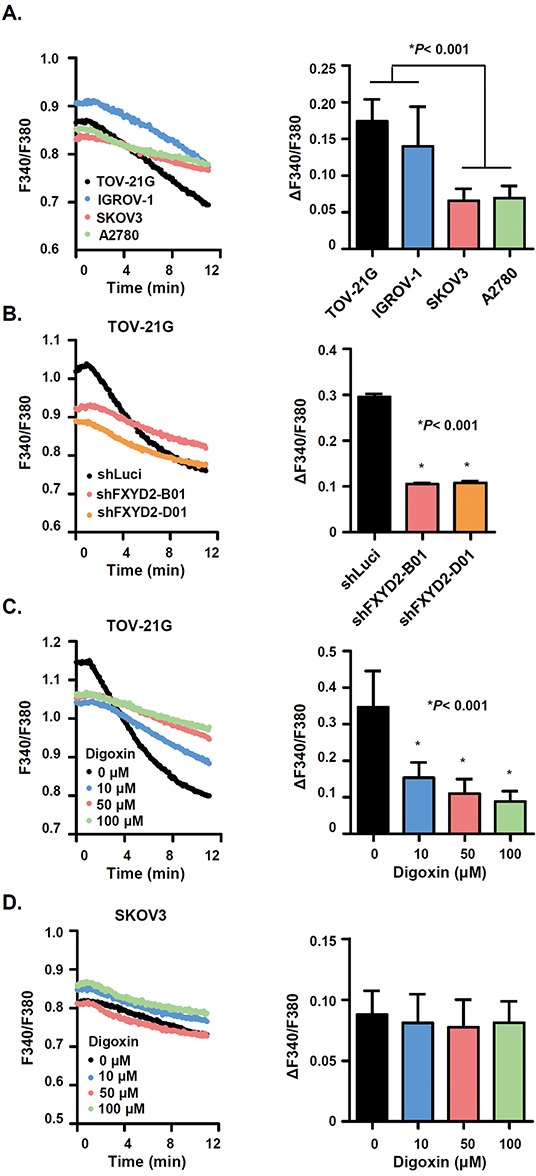
FXYD2 modulates Na^+^/K^+^-ATPase activity in OCCC cells **A.** Na^+^/K^+^-ATPase activity was assessed in ovarian cancer cell lines. Cells that were first blocked in K^+^-free buffer were then reactivated in Na^+^-free, 4 mM K^+^ solution. Na^+^/K^+^-ATPase was measured using a fluorescent indicator, SBFI. The F340/F380 changes (ΔF340/F380) following Na^+^ effluence in ovarian cancer cells were shown in the histogram. **B.** Na^+^/K^+^-ATPase activity was decreased in FXYD2-knockdown cells. **C.** and **D.** digoxin dose-dependently inhibited the Na^+^/K^+^-ATPase activity in OCCC but not in non-OCCC cells. Data are presented as the mean ± SD from 50 individual cells. All experimental groups were analyzed using an unpaired *t* test.

FXYD2 interacts with and modulates Na^+^/K^+^-ATPase activity by changing its affinity for Na^+^ and K^+^ [18]. Previous studies have indicated that the suppression of Na^+^/K^+^-ATPase expression or activity leads to the inhibition of breast and prostate cancer cell growth [16]. To verify the relationship between FXYD2 expression and Na^+^/K^+^-ATPase activity in OCCC, four ovarian cancer cell lines, including two OCCC cell lines with high FXYD2 expression (TOV-21G and IGROV-1) and two non-OCCC cell lines with low FXYD2 expression (SKOV3 and A2780), were used to analyze Na^+^/K^+^-ATPase activity. In OCCC cell lines, the decline in ΔF340/F380 fluorescence, which was used as a surrogate marker of Na^+^/K^+^-ATPase activity, was more obvious compared with the decline in non-OCCC cell lines (Figure 3A). When FXYD2 expression was silenced in OCCC cells, the slope of the fluorescence change became flat, and the Na^+^/K^+^-ATPase activity was significantly decreased (Figure 3B). These results validate FXYD2 as a modulator of Na^+^/K^+^-ATPase activity and suggest that FXYD2 expression correlates with Na^+^/K^+^-ATPase activity in ovarian cancer cells.

It had been suggested that cardiac glycosides, inhibitors of Na^+^/K^+^-ATPase activity, have anti-cancer effects. Therefore, we explored whether cardiac glycosides inhibit Na^+^/K^+^-ATPase activity in OCCC cells. Treatment of the high FXYD2 expressing OCCC cell line, TOV-21G, with the cardiac glycoside, digoxin, was shown to promote a dose-dependent decrease in the slope of fluorescence change (Figure 3C). In contrast, digoxin was not shown to inhibit Na^+^/K^+^-ATPase activity in the low FXYD2 expressing non-OCCC cell line, SKOV3 (Figure 3D). Together, our work suggests that the upregulation of FXYD2 may confer a cellular dependence on Na^+^/K^+^-ATPase activity and that the inhibition of Na^+^/K^+^-ATPase activity, either by FXYD2 suppression or by treatment with cardiac glycosides, may exhibit a therapeutic benefit in OCCCs with high FXYD2 expression.

### Cardiac glycosides inhibit OCCC cell growth via FXYD2

**Figure 4:**
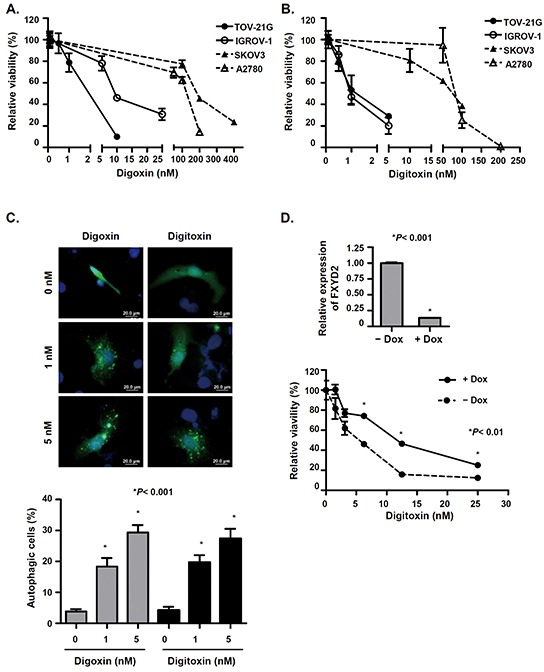
Cardiac glycosides suppress cell viability in OCCC cells in a FXYD2-dependent manner **A.** and **B.** cells were treated with various concentrations of digoxin or digitoxin for 48 h and cell viability was determined by WST-1 assay. **C.** cardiac glycosides induced autophagy in OCCC cells. TOV-21G cells were transfected with EGFP-LC3 plasmids and then treated with various concentrations of digoxin or digitoxin for 48 h. The cells with EGFP-LC3 spots represent autophagic cells. Cells were observed using a fluorescence microscope and counted among 200 EGFP-positive cells. Scale bar, 20 μm. **D.** knockdown of FXYD2 in OCCC cells decreased the efficacy of digitoxin-suppressed cell viability. TOV-21G cells were transduced with doxycycline-inducible shRNA against FXYD2. The expression levels of FXYD2 in cell with or without doxycycline were determined by qRT-PCR and normalized with GAPDH expression (upper). After pretreatment with 10 ng/ml doxycycline for 24 h, cells were treated with digitoxin for 48 h, and cell viability was determined by WST-1 assay (lower). Data from three independent experiments were analyzed using an unpaired *t* test.

Building on our findings that FXYD2 expression correlated with Na^+^/K^+^-ATPase activity, we next determined the effect of cardiac glycosides on the growth of OCCC cells. We found that cardiac glycosides efficiently inhibited ovarian cancer cell growth (Figure 4A and 4B). The IC_50_ values for digoxin for TOV-21G, IGROV-1, SKOV3, and A2780 cell lines were 4, 12, 216, and 101 nM, respectively. Additionally, the IC_50_ values for digitoxin for TOV-21G, IGROV-1, SKOV3, and A2780 cell lines were 2, 1, 68, and 84 nM, respectively. Surprisingly, the IC_50_ values for either digoxin or digitoxin for OCCC cell lines were lower than those for non-OCCC cell lines, and both digoxin and digitoxin inhibited OCCC cell growth at very low concentrations. These results indicated that OCCC cell lines with higher FXYD2 expression were more sensitive to cardiac glycosides compared with non-OCCC cells with lower FXYD2 expression. These results are consistent with our finding that the suppression of FXYD2 in OCCC cells or treatment with cardiac glycosides induced autophagy-related cell death (Figure 4C, Supplementary Figure S4). The Na^+^/K^+^-ATPase pump is composed of α and β subunits and the FXYD protein. Previous studies indicate that cardiac glycosides inhibit Na^+^/K^+^ ATPase activity by targeting the α subunit [20]. To evaluate which specific Na^+^/K^+^-ATPase subunit is involved in cardiac glycoside-induced cell death in ovarian cancer cells, we compared Na^+^/K^+^-ATPase subunit expression across several ovarian cancer cell lines with cardiac glycoside sensitivity. We showed that FXYD2 was the most differentially expressed subunit between OCCC cell lines and non-OCCC cell lines (Supplementary Figure S5A) and high expression of FXYD2 correlated with cardiac glycoside sensitivity. No significant relationship was observed between the expression of any other Na^+^/K^+^-ATPase subunits and cardiac glycoside sensitivity. Next, we examined whether sensitivity to cardiac glycosides may be modulated by FXYD2. Doxycycline-inducible shRNAs were used to knockdown FXYD2 expression in the OCCC cell line, TOV-21G. The endogenous FXYD2 level and proliferation rate between TOV-21G and derived doxycycline-inducible FXYD2-knockdown cells were almost the same (Supplementary Figure S5B). The results showed that repression of FXYD2 promoted increased resistance to digitoxin (Figure 4D) in TOV-21G cells. Furthermore, we also found that digoxin inhibited the enzymatic activity of Na^+^/K^+^-ATPases but not the expression of the subunits (Supplementary Figure S5C). Taken together, our results suggest that FXYD2 expression modulates sensitivity to cardiac glycoside treatment in OCCC.

### Cardiac glycosides have a potential therapeutic effect in OCCC

**Figure 5:**
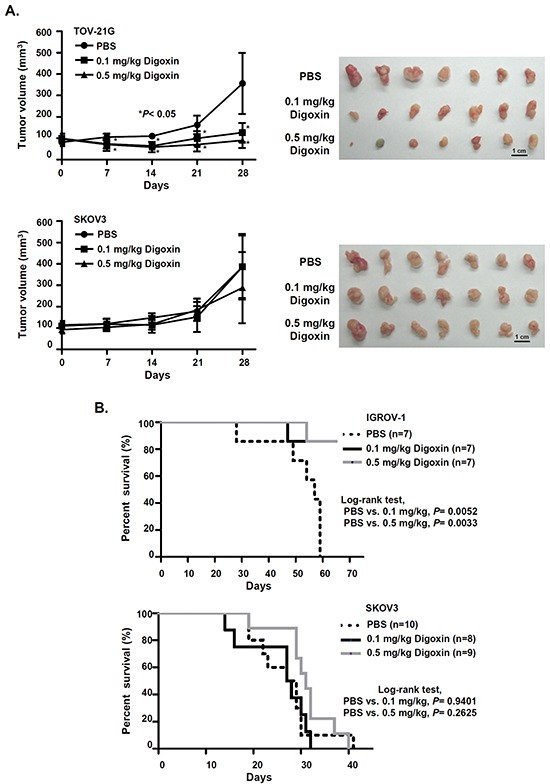
Cardiac glycoside significantly suppresses tumor growth and improves the survival of OCCC-bearing animals **A.** NOD/SCID mice were subcutaneously injected with 2.5×10^6^ OCCC (TOV-21G) or non-OCCC (SKOV3) cells (n = 7 mice per group). One week after inoculation, mice were daily intraperitoneally injected with PBS or digoxin for 28 days. Tumor volume was measured every 7 days. *P* values were determined using an unpaired *t* test. **B.** Kaplan–Meier survival curve of xenograft mice treated with PBS or digoxin. NOD/SCID mice were intraperitoneally injected with 5×10^5^ OCCC (IGROV-1) or non-OCCC (SKOV3) cells. Three weeks after inoculation, mice were intraperitoneally injected daily with PBS or digoxin. *P* values were determined using a log-rank test.

Our results showed that cardiac glycosides, digoxin and digitoxin, had great efficacy in inhibiting the cell growth of OCCC *in vitro*. Therefore, we asked whether cardiac glycosides also inhibited tumor growth of OCCC *in vivo*. In subcutaneous xenograft animal models, mice were intraperitoneally injected daily with PBS or digoxin for one month after tumors formed. The results showed that digoxin significantly delayed tumor growth of TOV-21G cells, but had no therapeutic effect on the growth of SKOV3 cells (Figure 5A). Similar results were also found in an orthotopic xenograft animal model. After ovarian cancer cells were injected into the abdominal cavity for 3 weeks, mice were given daily intraperitoneal administration of PBS or digoxin. We showed that digoxin successfully prolonged the overall survival in mice injected with IGR-OV1 cells but not with SKOV3 cells (Figure 5B). In conclusion, our results suggest that cardiac glycosides may be potential therapeutic agents for OCCCs with high FXYD2 expression.

### HNF1B directly regulates FXYD2 expression in OCCC

**Figure 6:**
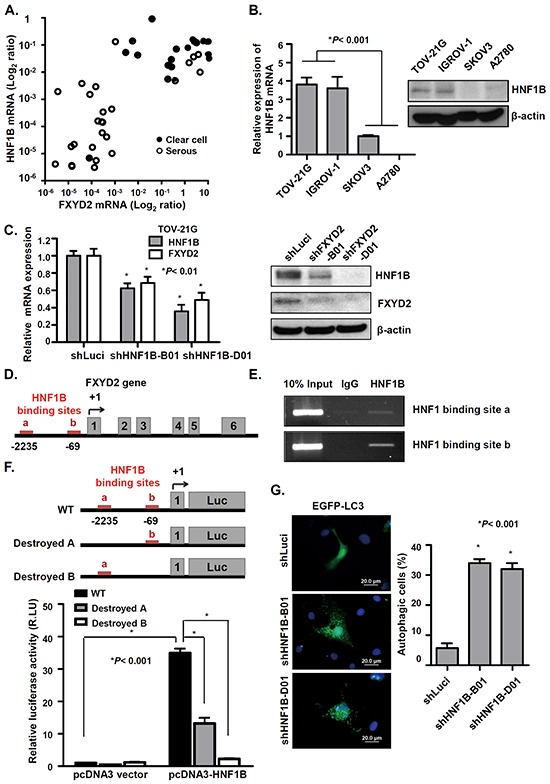
HNF1B transcriptionally regulates FXYD2 expression in OCCC **A.** the mRNA levels of HNF1B and FXYD2 in clinical ovarian cancer specimens were determined by qRT-PCR and normalized to GPADH expression. **B.** the endogenous HNF1B mRNA and protein levels in ovarian cancer cell lines were determined by qRT-PCR and Western blotting. β-actin was used as a protein loading control. **C.** HNF1B was knocked down in TOV-21G cells by lentiviral-based shRNA against HNF1B (shHNF1B-B01 and D01). The expression levels of HNF1B and FXYD2 were determined by qRT-PCR and Western blotting. **D.** the scheme of human FXYD2a gene (NM001680). Numbering of the nucleotides starts from the transcription initiation site. Red lines in the FXYD2 promoter region are two potential HNF1B-binding sites. Gray boxes represent the six exons of FXYD2a. **E.** chromatin immunoprecipitation analysis were used to evaluate the recruitment of HNF1B to the FXYD2 promoter in TOV-21G cells. Ten percent of total input DNA was used as a loading control and normal goat immunoglobulin G was used as an isotype control for the immunoprecipitation. PCR analysis was then performed using specific primers for the two potential HNF1B-binding sites in the FXYD2 promoter. **F.** Top: scheme of pGL3-FXYD2 promoter luciferase construct. Bottom: A2780 cells were co-transfected with a pGL3-FXYD2 promoter construct and pcDNA3-HNF1B expression plasmid. The luciferase activity was assayed in 24 hours later and normalized to the Renilla activity. The relative ratio of the luciferase activity to that in the vector control is represented as the mean ± SD. RLU: Relative Light Unit. **G.** HNF1B-knockdown cells were transfected with an EGFP-LC3 plasmid. Post 48-hour transfection, EGFP-LC3 spots were observed using fluorescence microscope. Scale bar, 20 μm. Cells with EGFP-LC3 spots represent autophagic cells and were counted among 200 EGFP-positive cells. All experiments were performed in triplicate and analyzed using an unpaired *t* test.

To further explore the molecular regulation of FXYD2 expression in OCCC, a computational approach and microarray expression profiling were used to predict possible transcription factors that regulate FXYD2 expression and transcription factor binding sites. Interestingly, we found that FXYD2 expression was positively correlated with the expression of a transcription factor, hepatocyte nuclear factor 1 homeobox B (HNF1B), in OCCC clinical specimens and cancer cell lines (Figure 6A and 6B). Previous studies demonstrate that HNF1B is a biomarker of OCCC [21, 22] and implicate HNF1B in the transcriptional regulation of FXYD2 in HEK293 cells [23]. To study whether FXYD2 overexpression in OCCC may occur via HNF1B, HNF1B was silenced using lentiviral-based shRNA in OCCC cells. HNF1B knockdown repressed the level of FXYD2 in TOV-21G and IGROV-1 cells (Figure 6C, Supplementary Figure S6A). Computational analyses predicted two binding sites of HNF1B in the promoter region of FXYD2 gene (Figure 6D). Next, whether the two possible HNF1B-binding regions at nucleotide (nt) -69 and nt -2235 in the FXYD2 promoter bound by HNF1B were tested using a chromatin immunoprecipitation assay. The results showed that both predicted binding sites for HNF1B in the FXYD2 promoter were indeed bound by HNF1B (Figure 6E). Subsequently, to verify that HNF1B regulates FXYD2 expression, the pcDNA3-HNF1B and pGL3-FXYD2 promoter constructs were co-transfected into A2780 cells with low levels of FXYD2 and HNF1B expression. The presence of exogenous HNF1B significantly increased the reporter activity up to 34.9-fold compared with that in the control vector group (Figure 6F). Importantly, reporter activity decreased when two HNF1B binding sequences were destroyed respectively. These data indicated that FXYD2 expression was directly regulated by HNF1B. Next, we tested whether knockdown of HNF1B also induced autophagy-related cell death. Similar to FXYD2 suppression, silencing of HNF1B in TOV-21G cells led to a significant increase in autophagosome formation and LC3-ll expression (Figure 6G, Supplementary Figure S6B). Together, these results demonstrate that FXYD2 upregulation in OCCC is directly mediated by HNF1B and that repression of HNF1B may induce autophagy-related death in OCCC cells with high FXYD2 levels.

## DISCUSSION

Ovarian cancer is a heterogenous disease. Among different types of epithelial ovarian cancer, OCCC represents an aggressive and chemoresistant subtype. Patients with OCCC have a high rate of recurrence and poor prognosis [2–4]. In the current study, we show that overexpression of FXYD2 drives high Na^+^/K^+^ pumping activity in OCCC cells and is predictive of poor disease-free survival of OCCC. High expression of FXYD2 in OCCC but not in other subtypes of epithelial ovarian cancers and normal ovarian tissues is attributed to the transcriptional upregulation of HNF1B. Suppression of FXYD2 or inhibition of Na^+^/K^+^ pumping activity by cardiac glycosides offers novel therapeutic implications for OCCC.

The homeostasis of membrane potential and osmotic equilibrium maintained by Na^+^/K^+^ pump is important for cell growth, differentiation, and cell survival [24, 25]. Our work demonstrates that FXYD2 (but not other subunits of the Na^+^/K^+^-ATPase) enhances Na^+^/K^+^ pumping activity and protects cells from autophagy-induced cell death in OCCC cells. However, the mechanisms of Na^+^/K^+^-ATPase mediated autophagic cell death remain unclear. Previous studies have shown cardiac glycosides induces autophagic cell death in non-small cell lung cancer cells through multiple signaling pathways including mTOR deactivation, ERK1/2 activation [26], and JNK activation [27]. JNK activation by cardiac glycoside causes a dissociation of Bcl-2/Beclin-1 complex, which following the release of Beclin-1 to induce autophagic cell death in NSCLC. In addition, Kouroku *et al*. have indicated that ER stress increases the LC3 conversion and the autophagic activity via the ATG12 upregulation [28]. It has been known that inhibition of Na^+^/K^+^ pump by cardiac glycosides may induce ER stress [29, 30]. Cardiac glycoside-induced intracellular Na^+^ accumulation causes an increased Ca^2+^ from the ER via Na^+^/Ca^2+^ exchanger. Therefore, cardiac glycosides treatment in Na^+^/K^+^ ATPase-unregulated cancer cells may enhance ER stress which eventually leads autophagic cell death. But the possible intermediates which are involved in Na^+^/K^+^ pump dysfunction induced LC3 conversion need to be further determined.

Here, we identified that high FXYD2 expression in OCCC was directly regulated by the transcriptional factor HNF1B. HNF1B has also been found overexpressed in OCCC and has been considered as a potential biomarker for OCCC [21, 22]. Additionally, Bowtell *et al*. have found increased DNA copy number of HNF1B in OCCC by array-based genome-wide measurement [31]. Shen *et al*. also showed that the CpG islands in the HNF1B promoter in clinical OCCC specimen are unmethylated, whereas they are found hypermethylated in serous type ovarian cancer [32]. Our results suggest that binding of HNF1B to the promoter region of the FXYD2 gene modulates the transcription of FXYD2 in OCCC. Therefore, we suggested that FXYD2 overexpression in OCCC might be due to HNF1B upregulation, either via DNA copy number gain or promoter demethylation. Although HNF1B has been considered as a potential biomarker that may promote growth advantage for OCCC [33], HNF1B also regulates many genes that are essential for physiological functions in normal cells [34, 35]. In addition, overexpression of HNF1B has been observed in atypical endometrial tissue and endometriosis and contributes to the differentiation of endometriotic epithelium to the clear cell lineage [36]. Thus, targeting HNF1B for cancer therapy may have severe side effects, and targeting downstream targets of HNF1B may be required. Our results suggest that the HNF1B downstream target, FXYD2, may represent a better therapeutic target for OCCC.

In our study, high expression of FXYD2 conferred high Na^+^/K^+^-ATPase activity and increased sensitivity to cardiac glycosides in OCCC cells. Cardiac glycosides effectively inhibited the tumor growth of subcutaneous OCCC xenograft and prolonged the survival of mice with an orthotopic xenograft. Besides being widely used to treat heart failure through activating cardiac myocyte hypertrophy and inducing vascular smooth muscle cell proliferation, cardiac glycosides have advanced to clinical trials as cancer therapeutics with variable success [37]. For example, breast cancer patients receiving digitalis have lower cancer incidence, tumors with a more benign histologic phenotype, and higher overall survival rate [13, 37, 38]. Patients with high plasma levels of digitoxin had decreased risk of leukemia/lymphoma and kidney/urinary tract cancers [14]. Inhibition of Na^+^/K^+^-ATPase by cardiac glycosides also can sensitize cancer cells to anoikis and can prevent distant metastasis [39]. Moreover, Shibuya *et al*. have shown that overexpression of Na^+^/K^+^-ATPase increases cancer cell sensitivity to cardiac glycosides [12]. Consistantly, we show that FXYD2 silencing drives OCCC cell resistance to cardiac glycosides. Hence, cardiac glycosides may provide OCCC patients with high expression of FXYD2, a more effective therapeutic option.

Furthermore, the implications for using cardiac glycosides in cancer therapy may be questioned concerning their potential to induce cardiovascular side effects. In this study, the IC_50_ of cardiac glycoside digitoxin or digoxin required to kill OCCC TOV-21G cells was 2 and 4 nM, respectively, which is similar with the therapeutic serum concentration of digoxin (0.5-2 ng/mL or 0.6-2.6 nM) [40]. Moreover, effective inhibition of tumor growth at lower concentrations might be observed after more prolonged exposure of tumor cells to cardiac glycosides. Taken together, we suggest that cardiac glycosides have potential therapeutic efficiency and selective anticancer effect in OCCCs with high FXYD2 expression.

In summary, our findings provide evidence for FXYD2 as a novel prognostic biomarker and a therapeutic target for OCCC. FXYD2 can mediate cancer cell growth by regulating the activity of Na^+^/K^+^-ATPase. Cardiac glycosides may be a potential therapeutic agent for OCCC with high FXYD2 expression.

## MATERIALS AND METHODS

### Patients

Ovarian cancer patients who underwent staging or cytoreductive surgery from January 2004 to January 2010 at the National Cheng Kung University Hospital (NCKUH) in Tainan, Taiwan were enrolled in this study. Clinical and pathology information, including age, stage, cell type and survival, was collected from medical charts and pathology reports. All staging met the criteria of the International Federation of Gynecology and Obstetrics Classification (FIGO). Histological classification was defined according to the classification standards of the World Health Organization. The medical records and pathological slides of the enrolled patients were used to determine clinical characteristics, pathological diagnoses, and outcome information. The clinicopathological features of 144 and 74 ovarian cancer specimens obtained from the NCKUH for immunohistochemistry staining and quantitative real-time PCR analysis, respectively, are shown in Supplementary Table S1 and S2. The research protocol and consent form were approved by the NCKUH institutional review board of the hospital, and written informed consent was obtained from each patient. Fresh tumor specimens were collected during surgery, and then immediately frozen in liquid nitrogen, and stored at −70°C until analysis.

### Human ovarian cancer cell lines

All of the human ovarian cancer cell lines used in our study, including TOV-21G, IGROV-1, SKOV3, and A2780, were obtained from the American Type Culture Collection (ATCC, Manassas, VA). TOV-21G cells were maintained in MCDB105 and M199 (1:1) supplemented with 15% fetal bovine serum (FBS, Invitrogen, Carlsbad, CA). The IGROV-1 cell line was maintained in RPMI1640 supplemented with 10% FBS. The SKOV3 cell line was maintained in DMEM/F-12 with 10% FBS. The A2780 cell line was maintained in RPMI1640 with 10% FBS, 0.1 mM MEM non-essential amino acids, and 10 mM sodium pyruvate. All of the culture media contained 5 mM L-glutamine and antibiotics. All of the human ovarian cancer cell lines were incubated at 37°C, in a humid 5% CO_2_ atmosphere.

### Measurement of Na^+^/K^+^-ATPase activity

The enzymatic activity of Na^+^/K^+^-ATPase was detected by using a single cell fluorometer and the fluorescent indicator SBFI, which measures intracellular sodium concentration. After limiting enzymatic activity by incubating cells in a K^+^-free buffer, cells were perfused with K^+^ buffer to reactivate the pump activity, and the fluorescence ratio (F340/380) of SBFI was used to measure stepwise changes in the intracellular sodium concentration. The slope of the fluorescent change (ΔF340/F380) was used as an indicator of Na^+^/K^+^-ATPase activity, and the fluorescent intensity was analyzed using the TILLvisION 4.0 (Till Photonics) program. At least fifty individual cells from three independent experiments were observed under various culture conditions.

### Animal models

All xenograft procedures were approved by the National Cheng Kung University institutional animal care and use committee and performed in 6-8-week-old female severe combined immunodeficient (NOD/SCID) mice. To examine the effect of FXYD2 silencing on tumor growth, cells expressing either a control shRNA (shLuci) or shRNA targeting FXYD2 (shFXYD2) cells were subcutaneously inoculated into the posterior flank of NOD/SCID mice. Tumors were measured once a week for two months with calipers to determine the length (L) and width (W), and the volume was calculated using the formula (L×W×W×0.45). To determine the anti-tumor activity of digoxin, we used xenograft and orthotopic animal models. In xenograft experiments, cells were subcutaneously injected into the posterior flank of NOD/SCID mice, and then mice were randomly assigned one week post-injection into groups that received either PBS or digoxin (0.5 and 1.0 mg/kg/day) daily via intraperitoneal injection for an additional 4 weeks. Tumor size was determined once a week. For orthotopic experiments, OCCC cells were intraperitoneally implanted into NOD/SCID mice, and then mice were randomly assigned three weeks after implantation into groups that received either PBS or digoxin (0.5 and 1.0 mg/kg/day) via daily intraperitoneal injection. Survival was assessed daily.

### Statistical analysis

Significant differences between data groups in clinical specimens were evaluated using one-way ANOVA followed by Bonferroni multiple comparisons test. Disease-free survival analysis was performed using a Kaplan-Meier curve and estimated by the log-rank test. Data from all three independent experiments were expressed as the mean ± SD and were then analyzed using an unpaired 2-tailed Student's *t* test. For all tests, *p* < 0.05 was used to define statistical significance. The results of immunohistochemistry staining were scored on the basis of the percentage of cells that displayed membrane-positive staining for FXYD2 (0 to 5%, low; > 5%, high). The relationship between FXYD2 expression and clear cell ovarian cancer was validated by a two-sided Fisher's exact test.

## SUPPLEMENTARY MATERIALS FIGURES AND TABLES


