# Supplementary material for: Targeting FXYD2 by cardiac glycosides potently blocks tumor growth in ovarian clear cell carcinoma
Source: Oncotarget. 2016 Feb 19;7(39):62925–38. doi: 10.18632/oncotarget.7497 (PMC5325337; doi:10.18632/oncotarget.7497)
Supplement: Supplementary file 1 [file oncotarget-07-62925-s001.pdf]

SUPPLEMENTARY MATERIALS AND METHODS

Chemicals

Digitoxin was purchased from Sigma-Aldrich (St. Louis, MO). For preparation of *in vitro* study, digitoxin was dissolved in dimethylformamide, and store at -20°C before use. Digoxin (LANOXIN®) was from GlaxoSmithKline (DSM Pharmaceuticals, Inc. Greenville, NC).

Quantitative real-time polymerase chain reaction (qRT-PCR)

Total RNA was isolated from clinical specimens or cell lines using TRIzol Reagent (Invitrogen) and reverse transcribed to complementary DNA using SuperScript™ III Reverse Transcriptase (Invitrogen) according to the manufacturer's instructions. All amplification reactions were performed using StepOne™ real-time PCR (Applied Biosystems, Foster City, CA) and the following TaqMan probes: FXYD2, Hs00242345_m1; HNF1B, Hs01001602_m1; and GAPDH, Hs99999905_m1 (Applied Biosystems). All reactions were performed in triplicate, and relative expression levels of mRNA were calculated using the delta cycle threshold method by subtraction of GAPDH expression.

Immunohistochemical determination of FXYD2

Formaldehyde-fixed tissue sections on the slides were deparaffinized. To retrieve antigen, tissue slides were autoclave boiled in 1 mM Tris-EDTA buffer for 30 min. Subsequently, tissue slides were incubated with 3% hydrogen peroxide for 10 min to inhibit endogenous peroxidase. The tissue slide were incubated with antibody against FXYD2 (ZW-5, Santa Cruz), diluted (1:50) in blocking buffer, at 4°C overnight in a wet chamber. Thereafter, the tissue slides were incubated with an anti-mouse/rabbit immunoglobulin G-horseradish peroxidase-conjugated secondary antibody for 1 hour at room temperature. The signal was detected using a DAB Substrate Kit (Zymed Laboratories). Finally, slides were counterstained with hematoxylin, and then dehydrated and embedded.

Gene repression by short hairpin RNA

Lentivirus vectors containing sequences encoding small hairpin (sh) RNA targeting luciferase, FXYD2 and HNF1B were purchased from the National RNAi Core (Academia Sinica, Taiwan). All lentiviruses were generated from HEK293T packaging cells in the RNAi service laboratory of NCKUH. The cell culture supernatant containing lentiviruses was harvested every 24 h until 72 h after transfection. Viral titers were determined by transducing HEK293T cells using diluted culture supernatants and tested by counting the number of viable cells after 2 days of culture in the presence or absence of antibiotics. Viral supernatants were stored at –80°C. The cells were infected with lentiviruses in the presence of 8 μg/ml polybrene (Sigma-Aldrich, St. Louis, MO). After lentiviral infection, recipient cells were incubated in fresh medium containing selective drug, puromycin for 48 hours. Puromycin-resistant cells were pooled for subsequent assays. The shRNA sequences used in this study are presented in Supplementary Table S3.

Cell proliferation, soft-agar colony formation, and focus formation assays

For cell proliferation assay, cells were planted into 24-well plate. Cells were trypsinized and counted after 24, 48, 72, and 96 hours incubation. Each time point represents the average of triplicate experiments. For soft agar colony formation assay, cells were seeded into 0.35% agarose on top of 0.5% agarose layer. After 4 weeks, colonies were stained with MTT reagent and fixed in methanol. For quantification, colonies were photographed and counted. For focus formation assay, 500 cells were seeded into 6-well plate. After 10 days incubation, foci were fixed in methanol, stained with 0.5% crystal violet, and then photographed and counted. All experiment groups were replicated 3 times.

Autophagy measurement

Cells were seeded into 6-well plates on glass cover-slips and allowed to attach overnight. The following day, cells were transfected with EGFP-LC3 plasmids using Lipofatamine 2000 (Invitrogen). After 24 h, the cells were fixed in 4% paraformaldehyde solution, permeabilized with 0.1% Triton X-100, and then mounted using antifade reagent with DAPI (Invitrogen). The slides were examined with immunofluorescence microscopy. GFP-LC3 dot formation was quantified by counting the number of cells with EGFP-LC3 spots among 200 EGFP-positive cells. All experiment groups were replicated 3 times.

Chromatin immunoprecipitation (ChIP)

Cells were cross-linked with formaldehyde, and the nuclei were isolated and digested using EZ-ZymeTM Chromatin Prep Kit (Millipore) to shear the DNA into fragments of 180 bp - 360 bp. The chromatin complexes were subjected to immunoprecipitation with 10 μg of antibodies against HNF1B (C-20, Santa Cruz), or normal goat IgG (Millipore). The immune complexes were collected using Magna ChIP™ Protein G Magnetic Beads

(Millipore) and were reversed by heating at 65 °C for 4 h. The DNA was purified with QIAquick PCR Purification Kit (QIAGEN) and was then amplified by PCR. PCR performed with GoTaq® Green Master Mix (Promega) was used to detect the HNF1B binding sequences. The primer sequences used in this study are presented in Supplementary Table S3.

Cytotoxicity assay

Cells were seeded into 24-well plates. After 18 hours incubation, cells were exposed to various concentrations of digitoxin or digoxin for 48 hours. Cell viability was measured by WST-1 assay (Roche) according to the manufacturer's instructions. The IC_{50} value resulting from 50% inhibition of cell growth was calculated by CalcuSyn program. Each point represents the average of triplicate experiments.

Western blot assay

Protein lysate was subjected to SDS-PAGE and followed by blotting to a PVDF membrane. The PVDF membrane was blocked with 5% skimmed milk, and then probed with anti-FXYD2 polyclonal antibody generated with the immunogenic peptide (CGGNKKRRQINEDEP), HNF1B (12A5.1, EMD Millipore), LC3 (APG8B, Abgent), Caspase 3 (Cell Signaling) or β-actin (Sigma-Aldrich) at 4°C overnight. Subsequently, the blots were incubated with appropriate horseradish peroxidase-conjugated secondary antibodies for 1 hour at room temperature. Specific proteins were detected using chemiluminescence with ECL Plus Western Blotting Detection Reagents (GE Healthcare Biosciences) and X-ray film.

Luciferase reporter assay

The luciferase reporter construct, pGL3-FXYD2 promoter, and pcDNA5/FRT/TO-HNF1B were purchased from Addgene. The putative HNF1B binding sequences in pGL3-FXYD2 promoter were destroyed by PCR and restriction enzyme digestion, and then inserted into pGL3 basic vector. Myc-tagged HNF1B was subcloned into pcDNA3.1 vector. pGL3-FXYD2 promoter plasmids or pGL3-FXYD2 promoter plasmids with destroyed HNF1B binding sequence, and pcDNA3.1-HNF1B plasmids were co-transfected into A2780 cells by using Lipofectamine 2000 (Invitrogen) according to the manufacturer's instructions. Renilla luciferase plasmids were co-transfected as a control of transfection efficiency. 24 hrs post transfection, firefly and Renilla luciferase activities were measured with the Dual-Luciferase® Reporter assay (Promega). Firefly luciferase activity was normalized to Renilla luciferase activity per well. All experiment groups were replicated 3 times.

SUPPLEMENTARY FIGURES AND TABLES

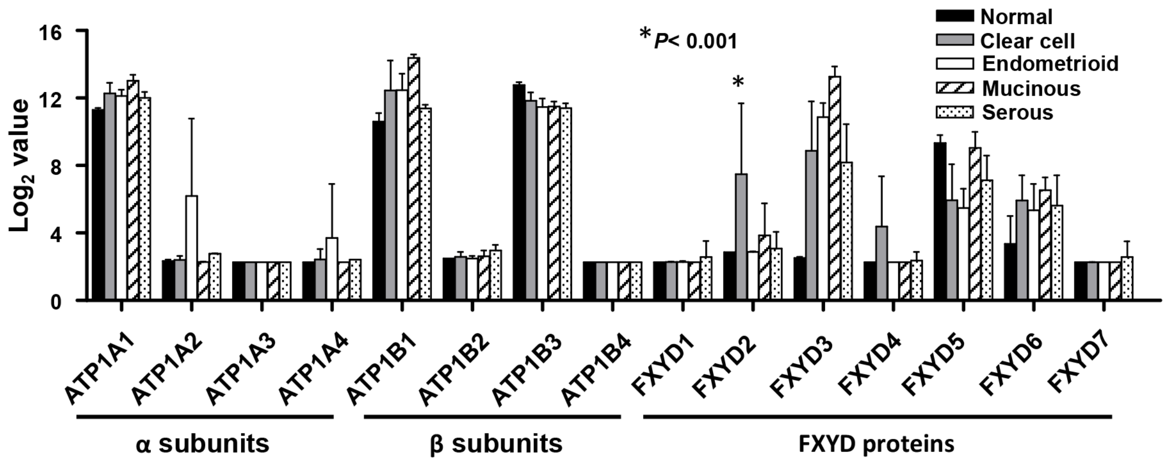

Supplementary Figure S1: FXYD2 is highly expressed in ovarian clear cell cancer. The $\log_2$ mRNA expression value of Na^+/K^+-ATPase subunits in clinical ovarian cancer specimens were determined by Affymetix GeneChip HG-U133_Plus_2 (GEO number: GSE44104). All of the specimen groups were compared to ovarian clear cell carcinoma group using one-way ANOVA followed by Bonferroni's multiple comparisons test.

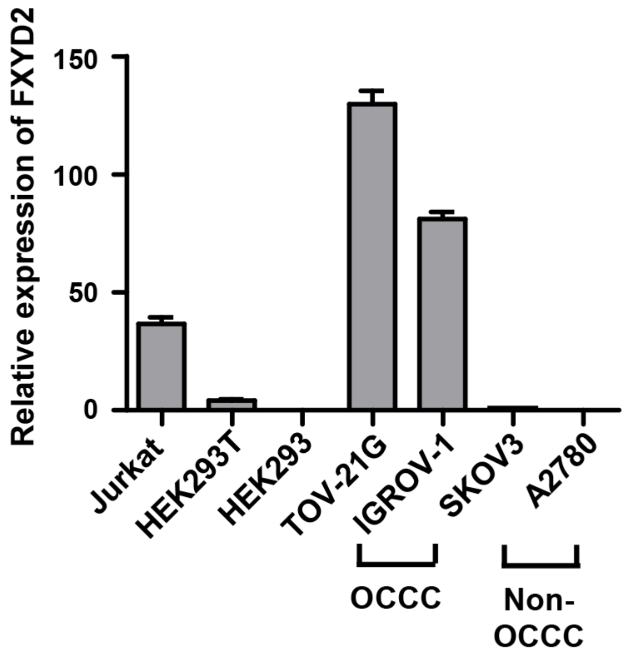

Supplementary Figure S2: FXYD2 is highly expressed in OCCC cell lines. The endogenous expression of FXYD2 in different ovarian cancer cell lines, human embryonic kidney cell line, HEK293 and derived cell line HEK293 T constantly expressing SV40 large T antigen. Jurkat cell line was used as positive control. FXYD2 mRNA levels were measured by qRT-PCR and normalized to the GAPDH expression.

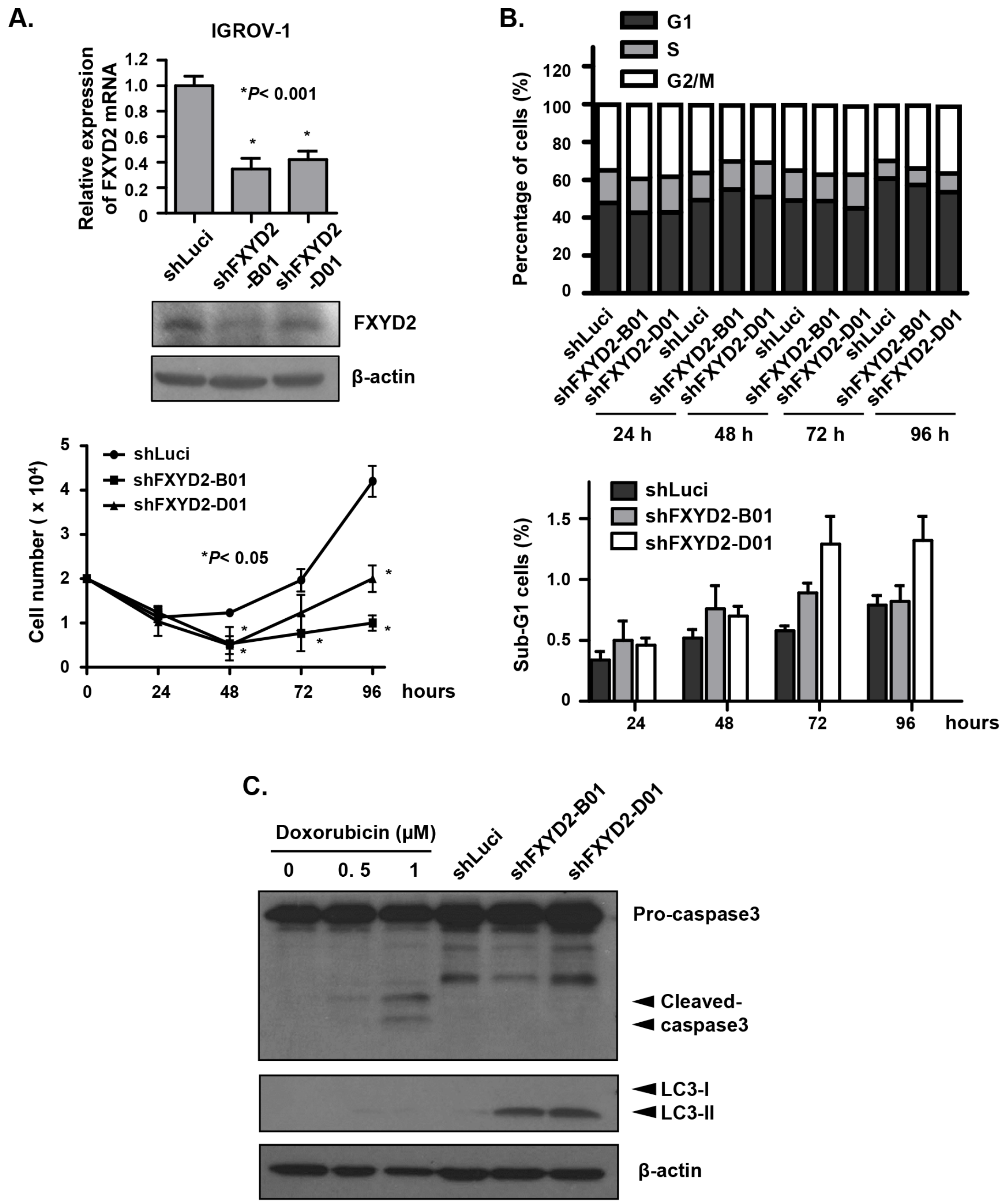

Supplementary Figure S3: Knockdown of FXYD2 inhibits proliferation of ovarian clear cell carcinoma. A. IGROV-1 cells were infected with lentiviruses carrying shB01 and shD01 against FXYD2 or the negative control luciferase (shLuci). IGROV-1 cells were grown in growth media for 24, 48, 72 and 96 hours. Cell proliferation was determined by cell number counting. FXYD2 mRNA levels were measured by qRT-PCR and normalized to the GAPDH expression. FXYD2 protein expression was determined by Western bloting. β-actin was used as a protein loading control. All of data from three independent experiments were analyzed using unpaired *t* test. **B.** TOV-21G cells were infected with shFXYD2 or shLuci viruses. Cells were starved in serum-free medium to induce cell cycle synchronization and then were released into complete medium for indicated times. Cells were collected, fixed, and stained with propidium iodide and subjected to flow cytometry to determine the distribution of cell cycle phases. Top, histograms show the proportion of shFXYD2 or shLuci infected cells corresponding sub-G1, G1, S, and G2/M phases. Button, the percentage of cells in sub-G1 phase was shown the mean and SD of three independent experiments. **C.** TOV-21G cells infected with shFXYD2 or shLuci viruses and the cell lysates were collected to detect cleaved-caspase3 and LC3-I/II expression respectively by Western blotting. β-actin was used as loading control. TOV-21G cells were treated with doxorubicin at the indicated concentrations for 48 hours and served as apoptosis positive controls.

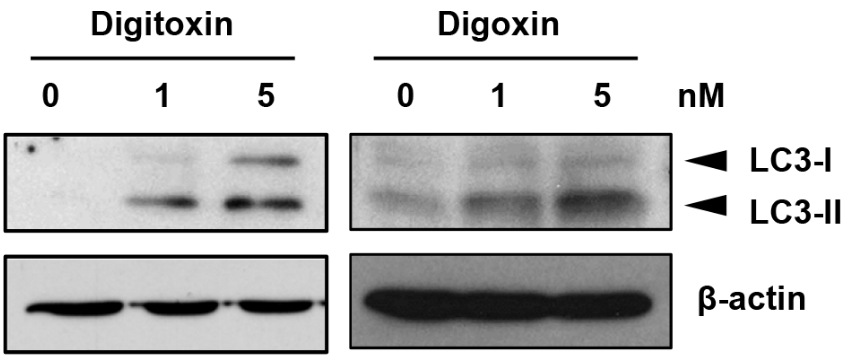

Supplementary Figure S4: Cardiac glycosides induce autophagy in OCCC cell lines. TOV-21G cells were treated with various concentrations of digitoxin or digoxin for 48 hours. The cell lysates were collected to detect LC3-I/II expression respectively by Western blotting. β-actin was used as loading control.

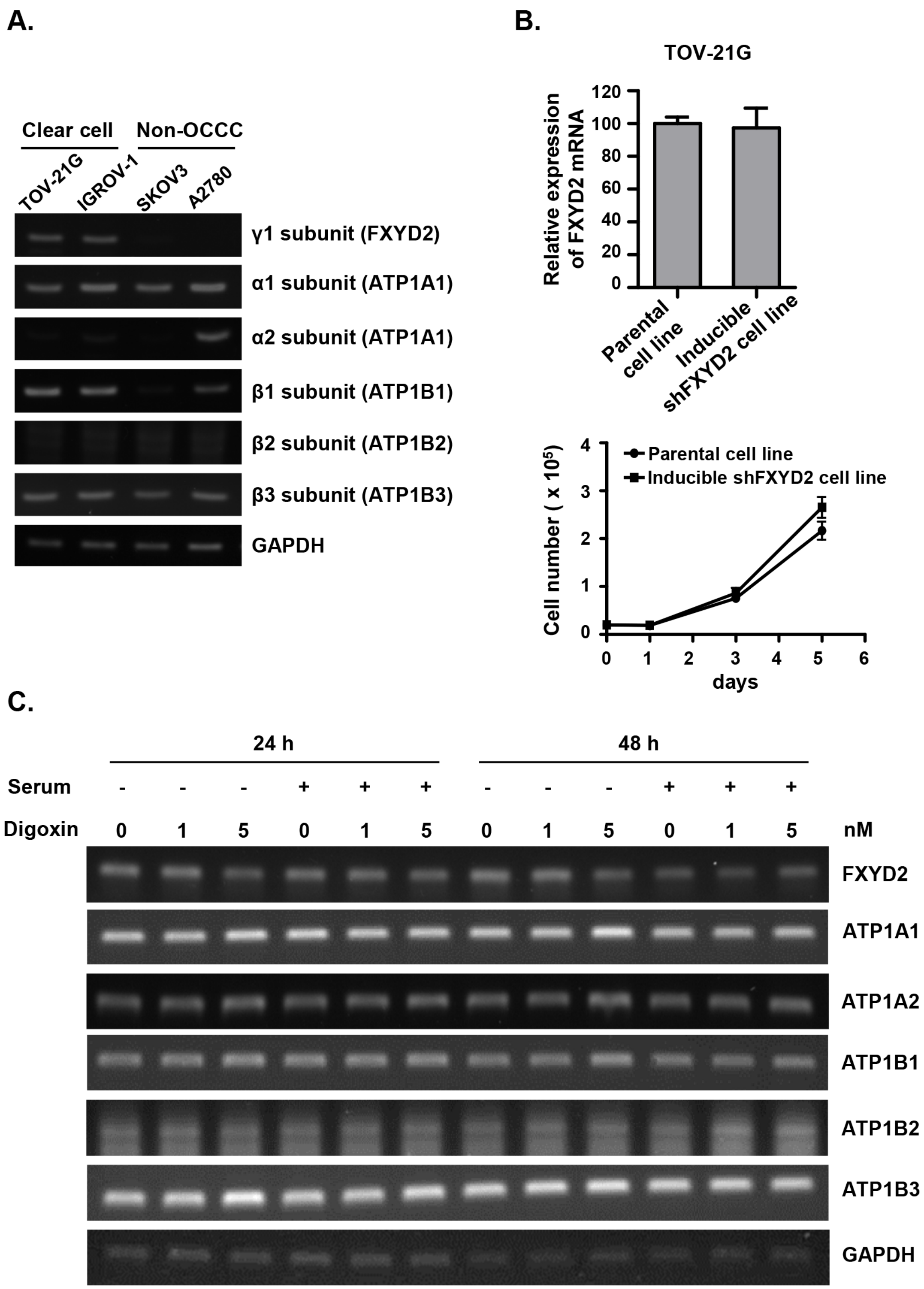

Supplementary Figure S5: Characterizations of ovarian cancer cell lines. A. endogenous levels of Na^+/K^+ ATPase subunits in ovarian cancer cells were determined by RT-PCR. GAPDH was used as an internal control. **B.** characterizations of TOV-21G cell line and derived doxycycline-inducible FXYD2-knockdown cell line. The endogenous FXYD2 mRNA levels were measured by qRT-PCR and normalized to the GAPDH expression. Cell proliferation was determined by cell number counting. All of data from three independent experiments were analyzed using unpaired *t* test. **C.** digoxin does not affect the expressions of Na^+/K^+-ATPase subunits in OCCC cells. TOV-21G cells were treated with various concentration of digoxin in serum-free or growth media for 24 and 48 hours. Expressions of Na^+/K^+-ATPase subunits were determined by RT-PCR. GAPDH was used as an internal control.

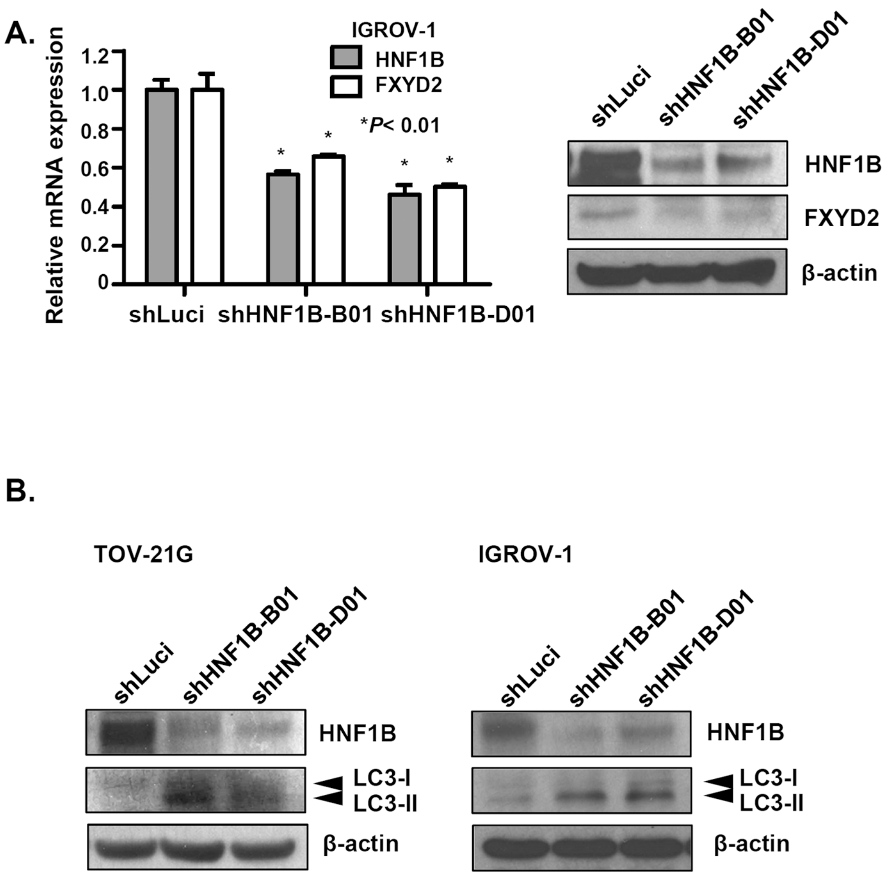

Supplementary Figure S6: HNF1B repression decreases FXYD2 expression and induces autophagy in OCCC cell lines. **A.** HNF1B was knocked down in IGROV-1 cells by lentiviral-based shRNA against HNF1B (shHNF1B-B01 and D01). The expression levels of HNF1B and FXYD2 were determined by qRT-PCR and Western blotting. **B.** TOV-21G and IGROV-1 cells were infected with shHNF1B- or shLuci-lentiviruses and the cell lysates were collected to detect LC3-I/II expression by Western blotting. β-actin was used as a loading control.

Supplementary Table S1: The correlation of FXYD2 expression and clinicopathological features of ovarian cancer specimens. Immunohistochemical staining of FXYD2 was detected in surgically resected specimens (*n* = 144).

	No. (%)	FXYD2 (+)	FXYD2 (-)	†P value
Age (mean ± standard deviation)				
54.19 ± 12.67 yr				
Histological type				
Clear cell	28 (19.44)	23	5	
Serous	70 (48.61)	6	64	
Mucinous	20 (13.89)	1	19	
Endometrioid	22 (15.28)	3	19	
Other cell type	4 (2.78)	0	4	
FIGO stage				
I	45 (31.25)	18	37	0.0404
II	10 (6.94)			
III	77 (53.47)	15	74	
IV	12 (8.33)			
Histologic type				
Clear cell	28 (19.44)	23	5	<0.0001
Other types	116 (80.56)	10	106	

† The association between FXYD2 expression and clinical features is shown in a 2 × 2 frequency table and validated by two-sided Fisher's exact test.

Supplementary Table S2: The correlation of FXYD2 expression and clinicopathological features of ovarian cancer specimens. qRT-PCR of FXYD2 was detected in surgically resected specimens (*n* = 74).

	No. (%)	FXYD2 (≥0.345)	FXYD2 (<0.345)	†P value
Age (mean ± standard deviation)				
51.59 ± 10.15 yr				
FIGO stage				
I	21 (28.38)	8	16	0.7934
II	3 (4.05)			
III	36 (48.65)	15	35	
IV	14 (18.92)			
Histologic type				
Clear cell	46 (62.16)	23	23	<0.0001
Serous	28 (37.84)	0	28	

† The association between FXYD2 expression and clinical features is shown in a 2 × 2 frequency table and validated by two-sided Fisher's exact test.

Supplementary Table S3: The sequences of primers for ChIP assay and RT-PCR, and shRNAs

Primer used in ChIP	Sequence (5' to 3')
HNF1 binding site a-F	CCACACTGCAGCTTTGTTTG
HNF1 binding site a-R	TTCCTGCTGTCTTTGCTCCT
HNF1 binding site b-F	AGCCCCTCCATCCACACT,
HNF1 binding site b-R	GTGTCTGGCTGCCTCCAC
**Primer used in RT-PCR**	**Sequence (5' to 3')**
FXYD2-F	ATGACTGGGTTGTCGATGGAC
FXYD2-R	CTACGGCTCATCTTCATTGATTT
ATP1A1-F	AAAGGTGTGGGCATCATCTC
ATP1A1-R	TGGTGACGGTGTGAATGACT
ATP1A2-F	TAAGGGAGTCATTGCCTTGG
ATP1A2-R	TTAGAAGCAGCGAGTGCATG
ATP1B1-F	GGACGTGCCCATTTTTACTG
ATP1B1-R	GCCAGTTTTATTCCCGTTGA
ATP1B2-F	GTCAGGCTGGTCTCGAACTC
ATP1B2-R	TGGGAAGTGGAACTTGAAGG
ATP1B3-F	TTGGTTGCTGTTCAGGTCAG
ATP1B3-R	CACCTTTCTCCAAGGTGGTC
GAPDH-F	TGGTGATGGAGGAGGTTTAGTAAGT
GAPDH-R	AACCAATAAAACCTACTCCTCCCTTAA
**shRNA**	**Oligo Sequence (5' to 3')**
shLuci	ATGTTTACTACACTCGGATATCTC
shFXYD2-B01	CCGGGACCCGTTCTACTATGACTATCTCGAGATAGTCATAGTAGAACGGGTCTTTTTG
shFXYD2-D01	CCGGCATCCTCCTCAGCAGAAGATTCTCGAGAATCTTCTGCTGAGGAGGATGTTTTTG
shHNF1B-B01	CCGGGCTGTTTCTCTTTCCAGAGTTCTCGAGAACTCTGGAAAGAGAAACAGCTTTTT
shHNF1B-D01	CCGGCCGACAATTCAACCAGACAGTCTCGAGACTGTCTGGTTGAATTGTCGGTTTT
